# Limbus Vertebrae as Incidental Finding in a Patient With Acute Lower Back Pain

**DOI:** 10.7759/cureus.10658

**Published:** 2020-09-25

**Authors:** Georgios Graikos, Antigoni Gkoudina, Nikolaos Tsakonas, Nikolaos Christakis

**Affiliations:** 1 Orthopaedics and Traumatology, General Hospital of Edessa, Edessa, GRC

**Keywords:** limbus vertebrae, interosseous herniation, spinal column, intervertebral disc degeneration, magnetic resonance imaging

## Abstract

Limbus vertebra (LV) represents a marginal interosseous herniation of the nucleus pulposus, usually in the midlumbar region. It is generally diagnosed as an incidental radiological finding during investigation of a case presenting low back pain or lumbar radiculopathy symptomatology. In this article, we report a case of a 37-year-old male patient complaining of acute low back pain. Imaging revealed a bone defect on the anterosuperior edge of L3 vertebra, finding that was attributed to LV. A comprehensive literature review was conducted regarding LV that should be included in the differential diagnosis of imaging findings of vertebral end defects.

## Introduction

Limbus vertebra (LV) is a condition characterized by marginal interosseous herniation of nucleus pulposus causing non-specific symptoms such as back pain, local muscle spasm and radiculopathy. It is frequently confused with vertebral fracture, infection, Schmorl nodule or tumor since it does not present any specific symptoms [1]. The characteristic appearance on plain films is represented as a detached fragment with triangular morphology and sclerotic margins, usually on the anterosuperior vertebral body corner. It represents a marginal herniation of the nucleus pulposus during childhood or adolescence through the vertebral endplate and beneath the apophyseal ring [2,3].

## Case presentation

A 37-year-old male patient presented to the emergency department complaining of sudden lower back pain after an abrupt rotation of the torso while playing football. He complained of sudden pain radiating from the lower back to the left lower limb without alleviation of the pain intensity during rest. No history of spine injury in the past was reported. During physical examination, no motor or sensory deficit was recorded. The Lasegue test, Achilles and patellar tendon reflexes were normal bilaterally. Lab tests were within the normal values.

**Figure 1:**
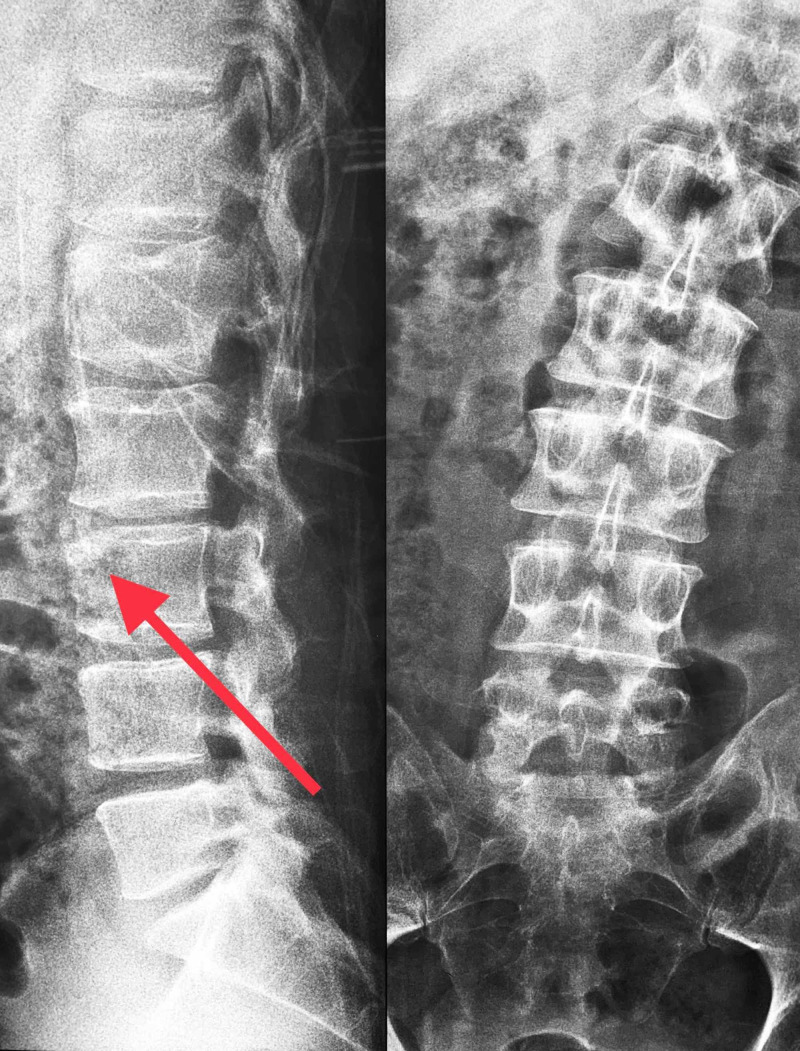
Irregularity along the anterosuperior edge of L3 vertebra with leftward antalgic scoliosis (at the site of sciatica).

On plain radiographs (Figure 1), a triangular bony fragment with sclerotic margins was demonstrated on the anterosuperior corner of the third lumbar vertebra.

**Figure 2:**
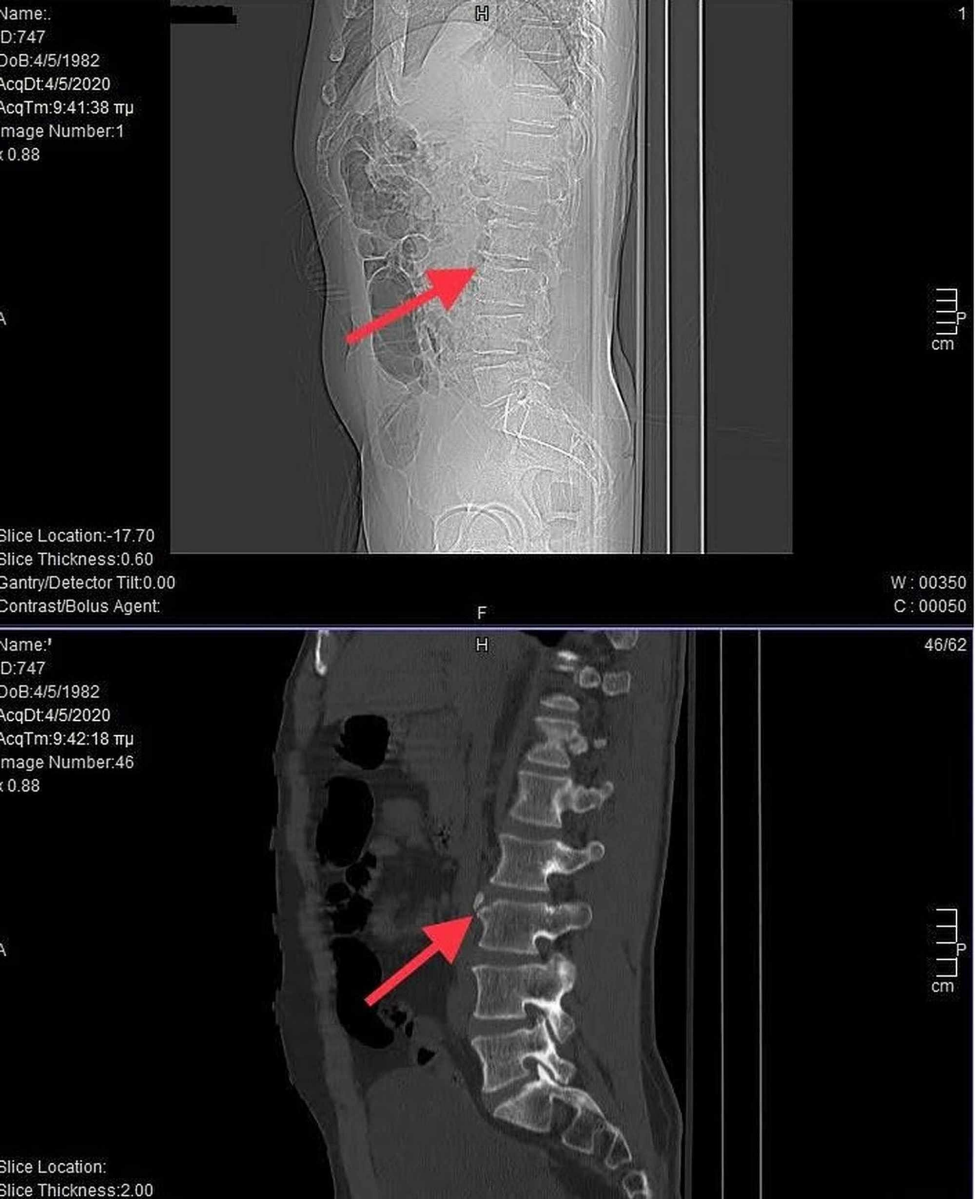
CT scan of lumbar spine showcasing a detached sclerotic triangular bony fragment on the anterior aspect of L3 superior endplate.

**Figure 3:**
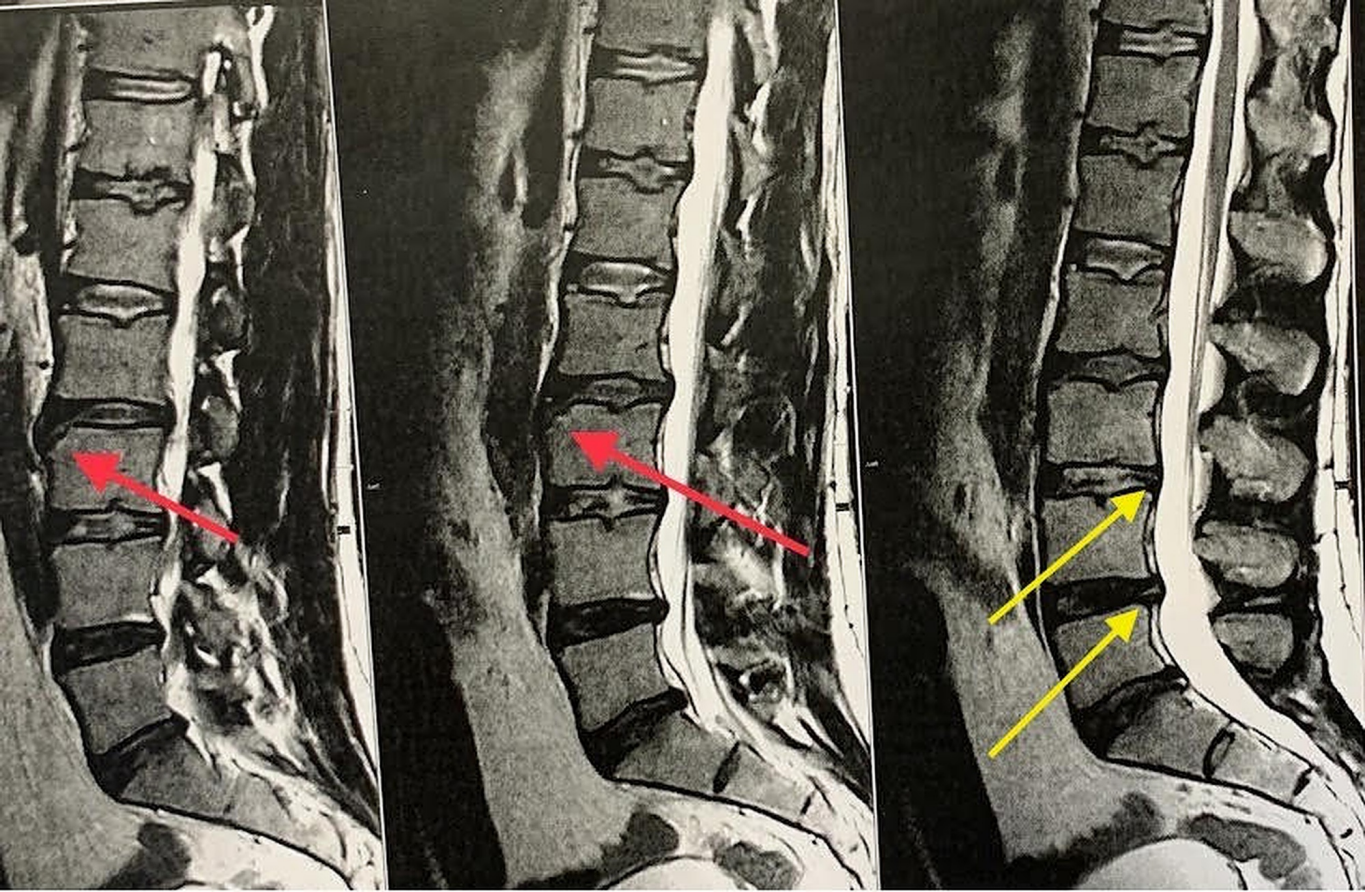
MRI scan, T2 sequence showing abnormal signal at the anterosuperior edge of L3 vertebra (red arrow) and adjacent intervertebral disc herniation at level L3-L4 and L4-L5 (yellow arrows).

Further testing was requested using CT (Figure 2) and MRI (Figure 3) lumbar spine evaluation since a more detailed approach was needed. It should be noted that MRI is useful to distinguish the LV lesion from an acute avulsion fracture of the anterosuperior corner accompanied by bone bruises. CT described a bony detachment along the anterosuperior edge of the L3 vertebra. The MRI examination confirmed the findings of the CT and showed a mild widespread protrusion of the intervertebral discs without compression effect at the level of L2-L3 and L3-L4, a central disc herniation in L4-L5 and a central disc herniation with an arising rupture of the annulus fibrosus compressing the thecal sac at the same level, producing the lower back pain symptomatology. Mild degenerative Modic changes on the last plates of L4 vertebra were demonstrated. A disturbance of the physiologic architecture of the anterosuperior part of L3 vertebra body attributed to LV was observed. 

The patient was admitted to the Department of Orthopaedic Surgery and treated conservatively. He was discharged the following day reporting obvious improvement of the symptomatology. 

## Discussion

LV displays a primary pathology of the intervertebral disc, occurring as a result of herniation of the nucleus pulposus into the adjacent vertebral body. When this process takes place during childhood and adolescence before ossification happens, the extruded disc material disrupts the endplate, warding off the ring apophysis from the vertebral body [3]. This condition was described for the first time by C.G. Schmorl in 1927 though its prevalence remains unknown [4]. LV can affect the anterior or posterior column of the cervical and lumbar spine, with the anterorsuperior margin of a single vertebral body of the midlumbar spine being the most common site of manifestation [5].

While the mainstream of patients leads an asymptomatic life with LV being an accidental finding in the lateral radiographic series of the spine, occasionally it can manifest with non-specific symptoms and signs, such as acute or chronic low back pain, spasm of the paravertebral musculature and radiculopathy, with or without trauma history [1,3,6]. Posterior limbus vertebra (PLV), although much more less frequent than anterior lumbus vertebra (ALV), can be symptomatic if it results in nerve compression mimicking an intervertebral disc herniation [7,8]. Recent studies demonstrated the involvement of TT genotype of COL11A1 polymorphism along with age and sporting experience being risk factors of LV [9]. Furthermore, intervertebral disc degeneration (IDD) seems to be related to ALV [10]. Among other apophyseal anomalies, LV represents the result of disc herniation into the adjacent vertebral body [11]. Numerous theories have been developed for the pathophysiology of this condition, which currently remains unclear. Koyama et al. described a strict correlation between TT COL11A1 genotype and LV in Japanese gymnasts while assuming that during growth sprout the epiphyseal plate is affected by a decreased expression of COL11A1 mRNA resulting in a weak epiphyseal plate [9,10]. Akhaddar et al. [12] supported that shear stress or trauma (hyperextension or concomitant violent flexion and axial compression of spine) during childhood and adolescence leads to a prolapsing nucleus pulposus before cartilaginous ring apophysis and vertebral body ossification completes.

The radiographic findings are typical in adults consisting of a small triangular or round bony fragment with sclerotic margins and osseous density. The adjacent vertebral body showcases a bony defect not corresponding completely to the later fragment with sclerotic and irregular borders. CT and MRI techniques can be used in cases where final diagnosis could not be established from plain radiographs and especially in difficult cases of PLV where lower lumbar vertebras are overlapped by pelvic structures [8].

Many pathologic conditions such as vertebral fractures (teardrop fractures), focal calcification of posterior or anterior longitudinal ligament, osteophytic fragments, calcified intervertebral disc hernias, tumors, infection and Schmorl’s nodules can mimic LV and must be excluded before setting the final diagnosis [3,13]. The presence of sclerotic borders of osseous fragment and the bone defect of the adjacent vertebral body help differentiate LV from an acute vertebral fracture in plain radiographs [3,6,14,15]. The size of bony detachment plays an important role in diagnosis since it cannot exceed the vertebral body’s defect [3]. CT has 100% sensitivity and specificity for identifying LV (especially PLV) in cases where plain radiography is not conclusive, depicting in detail the structure and location of osseous deficit and fragment in sagittal and coronal views [15]. The marginal herniation of LV aids in differentiation from Schmorl’s nodule which is represented by central herniation [5]. MRI, although not superior to CT concerning bony fragments, excels in revealing more detailed information about intervertebral disc and spinal canal pathology such as laminar erosions and intervertebral disc herniation with or without accompanying edema or inflammation [3]. Proton density sequence is reported to overbalance T1 and T2 images, highlighting small bony fragments, with gradient-echo sequence surpassing spin-echo [3,8]. Diagnosis can be puzzling when it comes to children since no typical radiographic findings are present, except for a focal irregularity of vertebral body [5].

When LV is an incidental finding and the patient does not present any symptoms, no treatment is required. As far as symptomatic patients are concerned, conservative management is the mainstay of treatment, including management with muscle relaxants, non-steroidal anti-inflammatory drugs (NSAIDs) and analgesics. Surgical management is rarely indicated and must be considered in cases of nerve compression causing radiculopathy resistant to conservative treatment. Total laminectomy is indicated for the excision of the LV fragment, while others support the removal of the mobile fragment alone [1,12].

## Conclusions

Despite LV being described almost a century ago demonstrating clear radiographic findings, the condition remains widely unknown to the medical community making the definite diagnosis challenging. A physician should include the LV variant in the differential diagnosis of an anterosuperior triangular well-corticated fragment of a vertebra in the midlumbar region in order to achieve a more immediate diagnosis and avoid inessential diagnostic procedures.

## References

[1] Tuna S, Özdemir T, Öz HE (2016). Limbus vertebra presenting with inflammatory low back pain: a case report. J Clin Diagn Res.

[2] Acosta-Ramón V, Pariente-Rodrigo E, Lara M, Pini SF, Rueda-Gotor J (2016). Limbus vertebra and chronic low back pain. J Fam Med.

[3] Kariki EP, Panagiotidoy D, Vasiliadis K, Theodorakopoulos A, Kotsabasopoulou I, Kotziamani N (2014). Limbus vertebrae: imaging investigation. Hellenic J Radiol.

[4] Espino-Rodríguez César A, García-Ballesteros Alejandra A, Castro-Prado Fernando C (1927). Uber die an den wirbelbandscheiben vorkommenden ausdehnungs-und zerreisungsvorgange und die dadurch an ihnen und der wirbelspongiosa hervorgerufenen veranderungen. Verh Dtsch Path Ges.

[5] Ghelman B, Freiberger RH (1976). The limbus vertebra: an anterior disc herniation demonstrated by discography. AJR Am J Roentgenol.

[6] Mupparapu Mupparapu, M M, Vuppalapati A, Mozaffari E (2002). Radiographic diagnosis of limbus vertebra on a lateral cephalometric film: report of a case. Dentomaxillofac Radiol.

[7] Sanal HT, Yilmaz S, Simsek I (2012). Limbus vertebra. Arthritis Rheum.

[8] Huang PY, Yeh LR, Tzeng WS, Tsai MY, Shih TTF, Pan HB, Chen CKH (2012). Imaging features of posterior limbus vertebrae. Clin Imaging.

[9] Koyama K, Nakazato K, Min S-K, Gushiken K, Hatakeda Y, Seo K, Hiranuma K (2012). COL11A1 gene is associated with limbus vertebra in gymnasts. Int J Sports Med.

[10] Koyama K, Nakazato K, Min S-K, Gushiken K, Hatakeda Y, Seo K, Hiranuma K (2013). Anterior limbus vertebra and intervertebral disk degeneration in Japanese collegiate gymnasts. Orthop J Sports Med.

[11] Hellström M, Jacobsson B, Swärd L, Peterson L (1990). Radiologic abnormalities of the thoraco-lumbar spine in athletes. Acta Radiol.

[12] Akhaddar A, Belfquih H, Oukabli M, Boucetta M (2011). Posterior ring apophysis separation combined with lumbar disc herniation in adults: a 10-year experience in the surgical management of 87 cases. J Neurosurg Spine.

[13] Şaş S (2019). Limbus vertebra mimicking avulsion fracture. Med Bull Haseki.

[14] Bağcıer F, Oğul H, Kul A (2015). Giant limbus vertebra mimicking a vertebral fracture. Turk J Osteoporos.

[15] Ross JS, Moore KR (2015). Diagnostic Imaging, Spine, 3rd ed.

